# A Cell Derived Active Contour (CDAC) Method for Robust Tracking in Low Frame Rate, Low Contrast Phase Microscopy - an Example: The Human hNT Astrocyte

**DOI:** 10.1371/journal.pone.0082883

**Published:** 2013-12-17

**Authors:** Alireza Nejati Javaremi, Charles P. Unsworth, E. Scott Graham

**Affiliations:** 1 Department of Engineering Science, University of Auckland, Auckland, New Zealand; 2 Centre for Brain Research, University of Auckland, Auckland, New Zealand; Tufts University, United States of America

## Abstract

The problem of automated segmenting and tracking of the outlines of cells in microscope images is the subject of active research. While great progress has been made on recognizing cells that are of high contrast and of predictable shape, many situations arise in practice where these properties do not exist and thus many interesting potential studies - such as the migration patterns of astrocytes to scratch wounds - have been relegated to being largely qualitative in nature. Here we analyse a select number of recent developments in this area, and offer an algorithm based on parametric active contours and formulated by taking into account cell movement dynamics. This Cell-Derived Active Contour (CDAC) method is compared with two state-of-the-art segmentation methods for phase-contrast microscopy. Specifically, we tackle a very difficult segmentation problem: human astrocytes that are very large, thin, and irregularly-shaped. We demonstrate quantitatively better results for CDAC as compared to similar segmentation methods, and we also demonstrate the reliable segmentation of qualitatively different data sets that were not possible using existing methods. We believe this new method will enable new and improved automatic cell migration and movement studies to be made.

## Introduction

Automated cell outline segmentation in live-cell time-lapse microscopy is of importance in a number of areas in cell biology such as: the assessment of cell culture quality, understanding the efficacy of drugs (on e.g. cancerous cells [1]), cell behaviour studies (such as scratch wound migration assays [2]) and in high-content screening for drug discovery [3]. Although live-cell fluorescence imaging has advanced microscopy considerably, the incorporation of dyes present issues with photo-bleaching and further complicate the long-timescale analysis of cells. One requirement of live cell imaging is that the cells are maintained at favourable biological conditions. To do this, cell illumination must be kept at a minimum. Phase contrast microscopy [4] is a technique that allows individual cells to be imaged at low light levels without the use of dyes, avoiding phototoxicity issues to a large extent. The drawback of this method is that it produces imaging artefacts such as halo and shade-off effects that complicate automated image analysis. 

While automatic segmentation methods for fluorescent microscopy are well-developed and in wide use [5] such methods are not as developed for phase-contrast microscopy [6]. 

**Figure 1:**
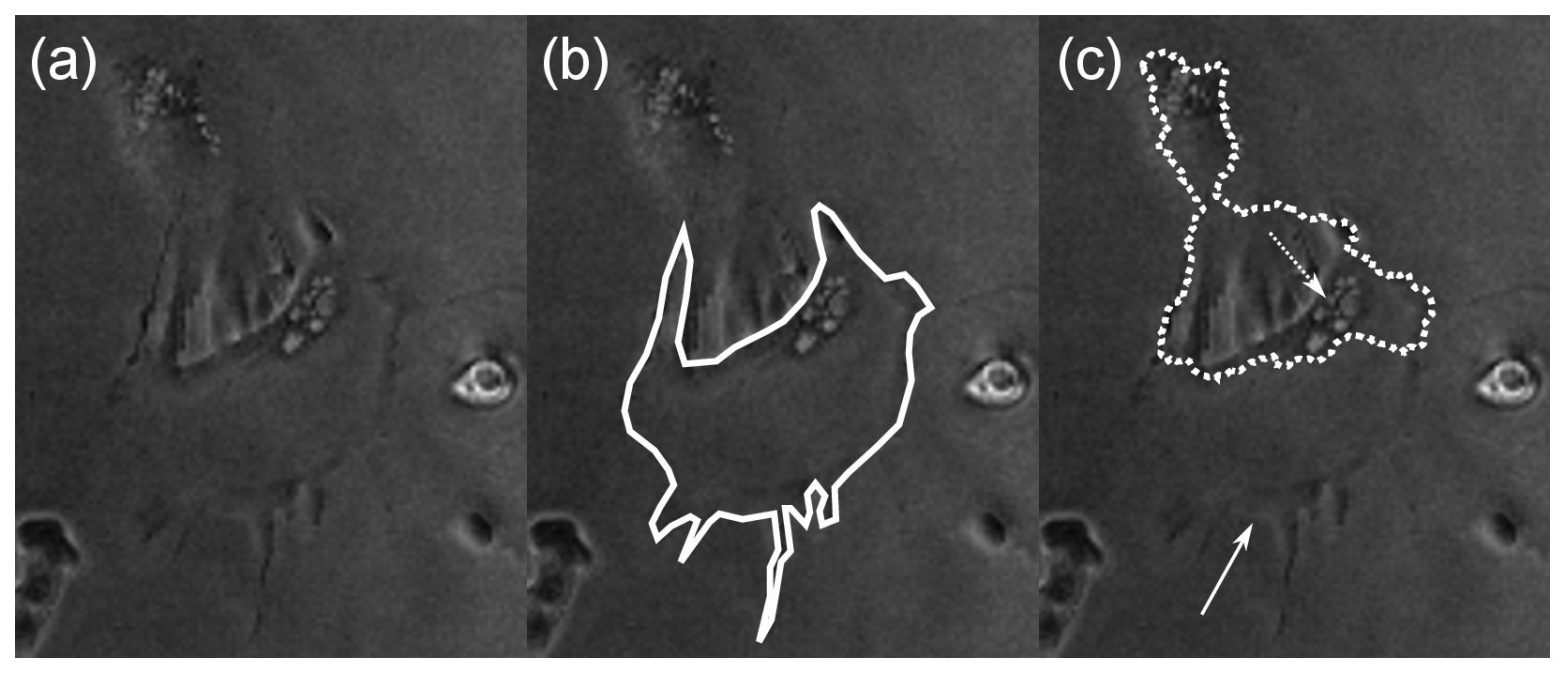
Low contrast and visual clutter impairs automated segmentation. a. An example of poorly-contrasted phase imagery, b. Expert-drawn segmentation. Note that the near-optimal thresholding procedure for automated segmentation (frame (c)) is very different from this and thus iterative refinement schemes might not work. The solid arrow indicates the cell boundary that should have been identified; the dotted arrow indicates the cell nucleus.

A difficult phase contrast segmentation problem and one that is often encountered is where the cell interiors have roughly the same brightness level as the image background (e.g. Figure 1) and the cell outline is not visible in many areas. Unfortunately, this is very common in phase-contrast microscopy as the end result of the phase-contrast optics is the attenuation of any information that lies outside a specific frequency band [7,8] (in the spatial domain, this is equivalent to convolving with the sum of an obscured Airy pattern and a dirac delta distribution).

Methods based on the watershed transform [9] have been used to some success on highly-contrasted phase imagery [10] but their utility is minimized in such low contrast situations (especially when local invisibility of cell boundaries is taken into account). Segmentation methods based on the statistical differences between cell interior and exterior [11] also have problems with data sets of this type. However, methods using supervised training seem promising for obtaining better performance [12,13]. Level set methods such as the Chan-Vese method [14] are region-based i.e. based on differences between the interior and exterior of objects and thus also have difficulty segmenting images with attenuated low-frequency information. For these types of problems, edge-based level sets, such as the magnetostatic active contour (MAC) model [15] and the geodesic active contour model [16,17], or combinations of geodesic and Chan-Vese level sets [18] are preferable.

Shape priors can be used to improve level set segmentation methods. One such form of prior is the multiphase level set method [19,20]. In this method, more than one level set function is used, and pixels are segmented based on the combination of level set interiors they belong to. A combined set of Mumford-Shah functionals is minimized to obtain the level set functions and thus the final segmentation. Various forms of geometric coupling forces [21] may be used to enforce certain shape patterns e.g. that nuclei can never extend outside the cell body, or that the width of the cell body must be over some threshold. In the region-based method of [21], it was shown how to combine different forms of geometric coupling with the Mumford-Shah functional minimization procedure. The multiphase method of [21] is highly suited to problems where the cell body, background, and nucleus are well-separated. Extending such methods to Mumford-Shah functionals containing edge-based terms dependent on the data might hold promise for segmenting phase-contrast imagery.

Video tracking methods, borrowing concepts from image segmentation and video compression, work well for tracking cells at high frame rates [6] even if contrast is low, but it is not clear how to extend these methods to e.g. time-lapse imagery, where cell boundaries may move significantly between frames, due to common sampling rates of less than one frame per minute.

Another consideration is that the use of automated microscopy setups permits the tracking of cells across a very large area, taking individual pictures of each area at each time step. While this greatly increases the available data, it reduces the frame rate since the microscope has to physically move to capture each frame. This prohibits the use of video tracking methods for such data sets.

Thus, the constraints that must be satisfied for a practical automated segmentation algorithm for live-cell phase microscopy are robustness to 1) High-noise, due to illumination constraints 2) Low contrast, due to cell thinness 3) Low frame rates, due to imaging hardware constraints.

Satisfying all of the above constraints seems to be a difficult problem. However, recent advances [22,23] have led to great improvements in automated cell tracking ability, even for phase-contrast imagery. For example, for the case of MDA-MB231 breast cancer cells with well-pronounced fronts, the Front Vector Flow Guided (FVF) snake proposed in [22] which is based on the gradient flow model [24] is able to provide segmentations for multiple cells. However, it is not clear how to extend the FVF algorithm to cells that do not have fronts i.e. polarizing or irregularly-shaped cells.

A notable advancement is the development of an active contour algorithm to both segment and track cells in phase-contrast microscopy using a 4-step level-set method; see 23. The method is a level set method similar to that first described in [14]. Variations of the level set method have been applied successfully to wide-ranging problems in computer vision, such as segmenting tomography images [25,26] and for finding aberrant crypt foci in the human colon [27]. There are three main factors that distinguish the Ambühl algorithm from previous methods. Firstly is a ‘near-optimal’ thresholding procedure that automatically locates the cells in the initial image using a completely automated procedure that attempts to find the ‘largest possible’ closed curves in the image (see reference for details). The second factor is that after a rough boundary has been found, it is refined using a top-hat filtered version of the image. This is done so that the located boundary is on the cell rather than on the phase-contrast halo – a problem that plagued many earlier active contour methods. The third difference, and the one most relevant to the discussion, is the emphasis put on the edges of the object. The energy functional to be minimized is designed so that the contour is driven towards one that has minimum length along a strong edge [23]. Thus the method is best described as an ‘edge-based’ method. Unfortunately, this introduces the problem that the initialization has to be relatively close to the edge to begin with (necessitating the non-level-set initialization procedure). In addition, Ambuhl's method suffers from lack of an iterative stopping criterion. These considerations hint that approaches using snakes rather than level sets might be more efficient, thus we used a snake-based method and we introduce a novel functional that is able to provide good segmentation and can be minimized globally and in a single step. Note that imposing either curvature regularity [28] or convexity constraints [29] leads to segmentation methods that can arrive at near-optimal initializations for further level set techniques, without requiring initialization. One concern for segmentation methods that do not rely on user-selected initializations is that the cell outline is often faint in comparison to other outlines (outlines of nuclei or debris in the image), thus it may not be easy to formulate an energy function that contains cell outlines as its *global* minimum but excludes those other outlines.

### Astrocytes

Our group is concerned with the accurate patterning of human neurons and astrocytes [30] on biocompatible materials fabricated on silicon chip for *in vitro* study. Thus, it is important to understand the migratory behaviour of such cells and how this behaviour is affected by the introduction of biocompatible materials. Our group also has interest in the markerless tracking of objects on the macroscale [31]. In this article, we develop markerless tracking methods to monitor the migratory movement of human brain cells at the micro-scale. The study of astrocytes [32] is particularly of interest, as in recent years, it has been uncovered that glia participate in many aspects of brain function [33]. During their development, astrocytes often migrate very long distances before `settling down' in a specific neural area [34]. To study astrocyte migration, our group has initiated research on how they can be guided to artificial structures [35,36,37]. In this study, we used a phase-contrast live cell imaging system with wide field of view (low magnification) to be able to track human hNT astrocytes moving over relatively long distances (up to several millimeters). It is of interest to be able to measure, in both ‘natural’ and artificial environments, the migratory distance travelled, phagocytic activity, and cell-cell interactions. Also of interest is to be able to precisely track fine dendritic projections which would aid in measuring complex behavior such as phagocytosis.

In this paper the tracking performance of the Ambühl method is applied to human astrocytes (differentiated from the NTera 2 stem cell line) [30] using time-lapse imagery. In addition, we present a method based on active contours called Cell-derived Active Contours (CDAC) to efficiently track cells over time.

We first explain the rationale behind the CDAC method, then examine why semi-automated methods (as opposed to fully automated segmentation algorithms) may be preferable for problematic data sets. We compare the results of CDAC with the Ambühl algorithm (and also 'baseline' reference parametric active contour method) on several data sets, highlighting domains that CDAC is better suited to, and showing that it can perform reliably and with less strict choice of parameters, requiring far less computational time. Especially, we stress the importance of finding ‘global’ optima, something that cannot be done efficiently with level set methods.

## Materials and Methods

### A. Cell culture

All media, serum and antibiotics were purchased from Invitrogen. The Ntera2/D1 cell line was purchased from ATCC. Astrocytes were produced using a 10–11 week differentiation protocol described in full previously [30,38](Burkert et al, 2011; Unsworth et al., 2011). In summary, the precursor stem cells are differentiated in the presence of 10 µM retinoic acid (RA) for 4 weeks, which is followed by various periods of treatment with mitotic inhibitors (MI; 10 µM cytosine arabino-side, 10 µM fluorodeoxyuridine (FUrd) and 10 µM uridine (Urd)(purchased from Sigma). Our protocol [38] produces both neuronal cells and astrocytes. The neuronal cells are harvested at week 6-7 during the mitotic inhibition phase. Typically, the initial neuronal harvest does not remove all of the neuronal cells [39], which grow on top of the differentiating astrocytes. It is important to remove as many of the neuronal cells, without loss of the astrocytic cells. At the end of the 10 week differentiation protocol, the hNT astrocytes were harvested by trypsinisation, counted and cryopreserved (in 10% DMSO, 40% FBS and 40% DMEM/F12) until required for experiments. 

### B. Microscopy

The Nikon BioStation™ IM live cell imaging platform was used. The cells were kept in a humidity, temperature, and CO_2_ regulated environment, and were periodically (once every 180 seconds) illuminated using a 620 nm LED light source and photographed using phase optics with 10× magnification. At this magnification level, the microscope produces images with 800×600 resolution, covering an area of 706×530μm. This is the resolution used by all our images.

### C. Image analysis

Raw data from the BioStation format (12-bit grayscale) were contrast-enhanced (without loss of information) and used for all processing. All processing was done on a Dell Optiplex 990 workstation with an Intel Core i5-2500 processor. The Ambühl method was run in MATLAB R2012a, the ABSnake method was performed in ImageJ and CDAC (see below) was implemented in C (all software single threaded). The Ambühl method took on average 410 seconds for 20 frames of the round cell (for the best run), 437 seconds for the extruding cell, 351 seconds for the retracting cell when in tracking mode and 707 seconds when in re-initializing mode. The corresponding times for CDAC were 1.1, 1.1, and 1.3 seconds for each of the cell types.

### The CDAC Algorithm

An optimal cell tracking algorithm would ideally outline the dynamics of the cells in question. Thus, in the developed algorithm we use *a priori* information about how cells move to aid the tracking process. In the following section, a brief introduction to the basics of cell motility is given, followed by a discussion as to how this information can be utilized to develop an efficient cell-derived active contour (CDAC) tracking algorithm.

#### A. Normal Extrusion

It is widely accepted that migration of metazoan cells occurs through the so-called ‘treadmilling’ action of actin [40,41,42,43]. Actin polymerizes into helices (known as fibrous actin or F-actin) that are distributed throughout the cell. These helices are polarized - they show different behaviour at either end. At the so-called ‘barbed’ end rapid polymerization takes place, but at the opposite end (called the ‘pointed’ end) polymerization is slow and hampered by the breaking apart of the polymer [44]. In addition, newly-attached monomers are tightly bound, but older monomers undergo a structural change (due to the breakdown of ATP) which causes the resultant helices to be unstable. For these reasons, the polymer appears to ‘move’ in a single direction.

If actin polymers are oriented randomly, no net movement occurs. The polymers simply extend until they reach the cell membrane where membrane and surface tension forces keep them in place. However, if the actin polymers are oriented along a single direction, migration may occur. Typically, in a migrating cell it is the case that the actin fibers in the cell are distributed in every direction, but near the lamellipodium they point preferentially to the moving front of the cell. The actin cytoskeleton is bound tightly to the substrate through cadherin and integrin adhesions [45,46]. Since the cell body is firmly attached to the substrate and the movement occurs by actin ‘pushing’ the membrane outward, a useful assumption is to assume that motility occurs by each point on the membrane extruding in the normal direction, either outward (at the cell front) or inward (at the trailing edge).

In addition, since the membrane is held tightly in shape by the actin cytoskeleton, it is not necessarily rounded by surface tension. On the contrary, migrating cells tend to show highly irregular and elongated shapes. For example, the lamellipodium itself has very high surface area to volume ratio. In addition, cells migrating in the mesenchymal mode [47] tend to produce long protrusions. Neurons produce growth cones that tend to form extremely long axonal projections or branched dendritic trees. Thus, it seems that a segmenting approach that does not penalise irregular snake shapes would be better suited to some cell types. These considerations lead to our active contour method.

#### B. Normal Extrusion Snake

While parametric active contours (snakes) are considered an ‘older’ method [48], we will show that they still provide much value for interesting segmentation problems. The active contour method considered in this paper is the one briefly described in [37]. A brief explanation follows. Let the snake be represented as $\hat{p}(s)=(x(s),y(s))$, (0 < *s* < 1). Given the image gradient **G**(r) (computed using a Sobel filter), the snake normal **N**, and total snake length **L**, the external energy is defined as:

$$E_{X}(\hat{p})=-\left|\frac{1}{L}\int_{0}^{1}\frac{1}{\left\|N(s)\right\|}N(s).G(\hat{p}(s))ds\right|$$ (1)

The contour that is used is piecewise-linear i.e. it is defined by a discrete set of sequentially-connected vertices ${\hat{p}}_{i}\;(1<i<n)$. 

**Figure 2:**
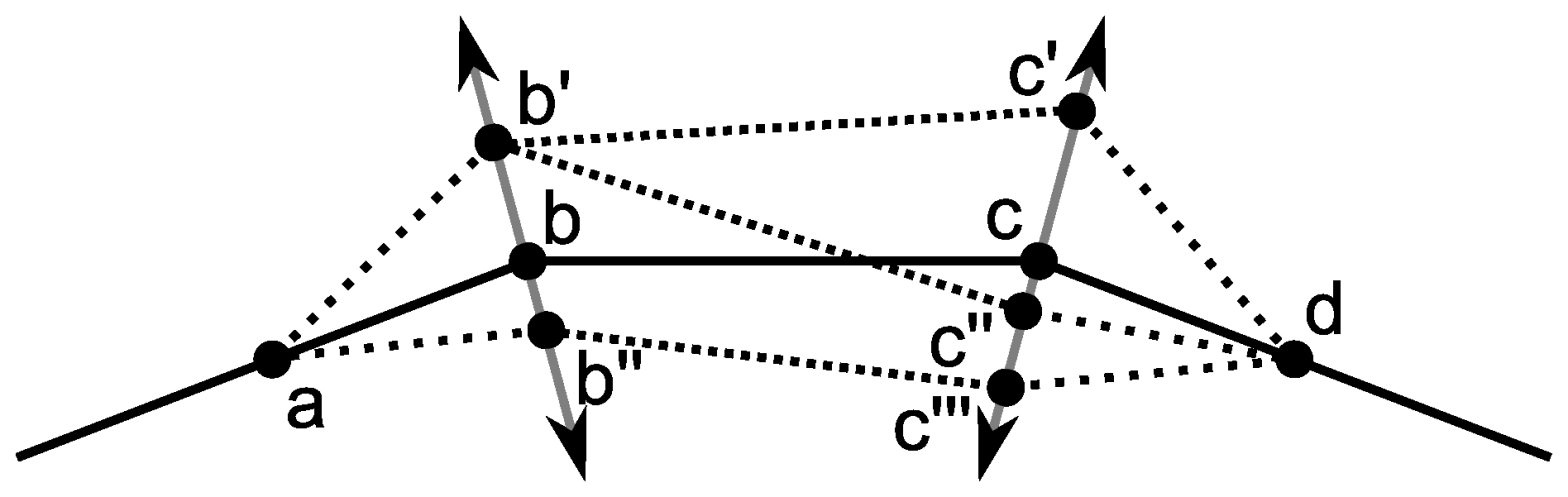
Normal extrusion procedure. The original contour is the solid line and a new contour may be produced by extruding, for example, points *b* and *c* by any amount.

Given an initial contour (e.g. from the previous frame), the vertices may be extruded to better fit the shape (Figure 2). The extrusion direction is computed as follows. Consider the *i*th point on the curve. The tangent vector at that point, for an extrusion level *x*, is given by:

$$T_{i}(x)=\sum_{j=i-M(x)}^{i+M(x)}e^{-c_{ij}{}^{2}/2x^{2}}\mathrm{sgn}(i-j)({\hat{p}}_{i}-{\hat{p}}_{j})$$ (2)

Where *M*(*x*) is chosen to be large enough that the exponential decays to zero. In our implementation, a 3-sigma radius is used i.e. *M*(*x*) = floor(3*x*). The contour itself is assumed to ‘wrap around’. In other words, ${\hat{p}}_{i}={\hat{p}}_{i+n}$ where *n* is the number of points on the contour. In addition, *c*_*ij*_ is the cumulative distance from the *i* ^th^ point to the *j* ^th^ point:

$$c_{ij}=\sum_{k=i}^{j-1}\left\|{\hat{p}}_{k}-{\hat{p}}_{k+1}\right\|$$ (3)

The normal N_*i*_ is simply chosen to be the unit vector forming a 90 degree angle with T_*i*_. We call the vector [11] of one extrusion level per pixel an *extrusion*. Note that there must be a limit *R* to the range of *x*_*i*_. We arbitrarily chose a value of 16 for *R*. Since the method usually does not select an extrusion level larger than a few pixels, this level was deemed to be usually sufficient. Indeed, since the level must conform to the movement of the cell boundary, selecting a large level is usually a tracking error and can be seen in the output that CDAC produces. 

The energy of the curve is minimized using a dynamic programming approach similar to that of [49,50]. This enables an efficient calculation of the optimum which cannot be done using level set methods and as will be shown is necessary for segmentation where edges are very weak and can only be non-ambiguously recognized holistically. A notable difference between the method given in the cited papers and the method given here is that here the neighborhood of each vertex is relatively large (16 pixels). This is possible due to the fact that the vertex is only allowed to extrude in one dimension (the normal direction). This allows us to explore a much larger space at each step. In addition, in contrast to most other snake methods, the snake method given here has no internal energy. This allows highly irregular cell shapes to be tracked.

The dynamic programming approach is as follows. Let the candidate extrusion levels be *x*_*i*_, where *i* ranges over the vertices on the contour. *x*_*i*_ is the the distance that each vertex extrudes or retracts to fit the contour in the next frame and is between –*R* and *R*. Also, let the multi-scale normal vector at each vertex be N_*i*_(*x*_*i*_) (the unit vector perpendicular to T_*i*_(*x*_*i*_) in equation 2). Here, ‘multi-scale’ refers to the fact that N_*i*_(*x*_*i*_) is not just dependent on *i*, but also the extrusion level *x*_*i*_.

The goal is to find the vector [*x*_1_, *x*_2_, ...,*x*_n_] that minimizes $E_{X}(\hat{r})$ where $\hat{r}$ is the piecewise-linear curve defined by ${\hat{r}}_{i}={\hat{p}}_{i}+x_{i}N_{i}(x_{i})$, and *x*_i_ is any integer in [-*R*, *R*]. For each pair of adjacent vertices p*^*_*i*_ and p*^*_i+1_, consider the (2*R*+1)^2^ line segments between r*^*_*i*_ = p*^*_*i*_ *+x*_*i*_N_*i*_(*x*_*i*_) and r*^*_*i + 1*_ = p*^*_*i + 1*_ *+x*_i + 1_ N_*i* + 1_(*x*_*i* + 1_), defined by varying *x*_*i*_ and *x*_*i* + 1_. Associate to each line segment its energy calculated according to the term *inside* the absolute value in equation 1. The minimization problem is then simply the problem of finding a minimum-cost path from any r*^*_0_ to any r*^*_*n*_, where negative costs may occur. This can be solved using the Bellman-Ford algorithm. To take into account the fact that *E*_*X*_ is defined as an absolute value, simply perform the minimization step twice, once negating the signs, picking the minimum amongst these two minima.

To control the amount of extrusion, an additional dynamic extrusion penalty κ is included in the energy. κ is defined as follows: If a line segment moves at all from its previous location (i.e. the extrusion on either of the two vertices it connects is nonzero), κ is added to that line segment’s energy. Otherwise, it is left unmodified. This allows one to restrict the curve from varying too much between frames; a high κ value will tend to keep the curve ‘inert’. Since the external energy can by definition never be larger than the maximum gradient magnitude in the image, any value of κ that is significantly higher than this would keep the curve inert. Thus the final energy function is:

$$E_{X}({\hat{p}}^{\left(t+1\right)})=-\left|\frac{1}{L}\int_{0}^{1}\frac{1}{\left\|N(s)\right\|}N(s).G({\hat{p}}^{\left(t+1\right)}(s))ds\right|-\kappa \left|S({\hat{p}}^{\left(t+1\right)},{\hat{p}}^{\left(t\right)})\right|$$ (4)

Where *S* (the set of line segments that have changed from the previous frame) is defined as follows:

$$S(\hat{p},\hat{q})=\{(p_{i},p_{i+1})|p_{i}\neq q_{i}\vee p_{i+1}\neq q_{i+1};i=1\;\mathrm{Kn}\}$$ (5)

The linear nature of *E*_*X*_ makes the described global optimization procedure possible, and that non-linear terms such as an internal energy based on curvature would disrupt this possibility.

We refer to our developed method as Cell-derived Active Contour (CDAC). In the following, CDAC is compared with the Ambühl method, for both synthesized data and for data from actual microscope set-ups. In addition, a comparison is also done between CDAC and the standard ‘baseline’ gradient-descent snake implementation given in as follows: the edges are found using a Canny-Deriche operator [51,52] and internal energy is computed from the curvature of the snake:

$$E_{I}(v)=\int_{0}^{1}\alpha {\left\|v^{\prime }(t)\right\|}^{2}+\frac{1}{2}\beta {\left\|v^{\prime \prime }(t)\right\|}^{2}dt$$ (6)

Such snakes have been used widely in many image-processing tasks [53,54,55]. One limitation of these snake algorithms is that they require fine-tuning of the many parameters involved. To get around this limitation, an adaptive snake algorithm was proposed [56]. In this method, the parameters of the model are varied automatically at each step to produce the best fit. This algorithm is available as a plugin (ABSnake) for the image-processing tool ImageJ–a popular tool used frequently by researchers in microscopy and cellular biology

#### C. Segmentation vs. Tracking

All active contours are, by nature, capable of tracking - simply set the initial contour for the next (current) frame equal to the contour for the previous frame. However, there must be a way of initializing the contour for the first frame. In the Front Vector Flow (FVF) method of Li et al [22] (described in the section ‘Introduction’), the initial segmentation is provided by localizing the cell nucleus using a combination of Laplacian-of-Gaussian (LoG) filters and fuzzy c-means clustering [57]. This is applicable to cells where the nucleus is highly contrasted against the background but it is not clear if it would work well otherwise. In the Ambühl method, the initialization is provided by a near-optimal thresholding procedure. A drawback of this procedure is that it is susceptible to noise and visual clutter (Figure 1). When automatic segmentation procedures fail, the contour has to be initialized manually.

It is worth noting that if a particular data-set is such that a fully automatic segmentation procedure produces segmentations of quality comparable to expert-drawn outlines, then it is desirable to produce a new segmentation for each frame rather than initializing from the previous frame. This is because in the latter case, segmentation errors will tend to accumulate [53]. 

Since CDAC relies on the property of cells extruding normal to their boundaries, it is not capable of converging from a very ‘rough’ outline, unless the cell outline can be obtained from the rough outline by means of normal extrusion. This is unlike other snake methods. However, once a ‘lock’ is obtained, CDAC is good at maintaining it, as will be demonstrated.

Thus, to make a fair comparison, the comparisons are done on data sets with high noise where the initial contour segmentation procedure fails. This allows us to initialize both methods (both the Ambühl method and CDAC) to the same contour and follow the cells as they migrate around the sample.

#### D. Differences from previous Snake methods

In summary, the method thus presented is different from most previous snake methods. It uses gradient direction instead of magnitude and the energy is normalized over length. This prevents from penalizing irregular shapes and allowing ‘tight’ fits to the contour to be obtained. In addition, self-intersections are automatically removed. Finally, normal extrusion and global optimization are used, allowing a far more efficient search of the possible curve space. While most of the attributes in CDAC have been seen in some form in other methods, we are not aware of any previous methods that have used all these attributes together.

## Results

In the following sections, the three methods (CDAC, the Ambühl method, and ABSnake) will be compared on three different data sets of increasing complexity. The first data set (the simplest) consists of a simple round cell that does not move rapidly. The second data set is a stationary cell with extrusions, and the third (most complex) is a cell with thinner extrusions and higher motility that also interacts with another cell.

A quantitative measure for assessing segmentation performance is required. In Ambühl’s method [23] an area-based metric is used – the Dice coefficient – which is the proportion of correctly-classified pixels. The definition is as follows. Let the set of pixels enclosed by the test contour be T and those enclosed by the reference contour be R. The dice coefficient is computed as 2|*R*∩*T*|/(|R|+|T|). For irregularly-shaped cells, especially those with narrow protrusions, this is not an adequate metric. However, it is the metric used in [23], and thus to provide a fair comparison with Ambühl’s method it is the metric that is used. Outline-based metrics such as the Hausdorff distance are better-suited to the cell shapes given in this paper, but to apply them to level set methods (especially those that can give multiple boundaries), several simplifying assumptions are required which complicate the analysis.

In addition to this, the sensitivity and specificity of the methods were quantified. The sensitivity is defined as TP/(TP + FN) and the specificity is defined as TN/(TN + FP). TP is the number of pixels inside the boundary that have been correctly classified as being inside the boundary. TN is the number of pixels outside the boundary that have been correctly identified as such. FP is the number of pixels outside the boundary that have been classified as being inside the boundary, and FN is the opposite of this (the number of pixels inside that have been classified as being outside).

For the initial frames, the user-defined boundary provides a significant bias that skews the results towards high sensitivity and selectivity. Since the reference cell is the same as the user-defined contour in the initial frame, and neither the test nor reference images deviate much from the previous frame, in the first segmentation frame the sensitivity and specificity are both close to 100% in the first frame. Thus, to evaluate the method as best as possible, the sensitivity and specificity should ideally be calculated excluding the first frame. The sensitivity and specificity are given in Table 1.

**Table 1:** Parameters for the Ambühl method, along with results.

**Data series**	**Parameters (A)**	**Parameters (B)**	**Sens (CDAC)**	**Sens (Ambühl)**	**Sens (ABSnake)**	**Avg (CDAC)**	**Avg (Ambühl)**	**Avg (ABSnake)**
Round cell	100 10 100	100 0.1 50	98, 98	97, 98	88, 95	0.94±0.03	0.95±0.03	0.87±0.08
Extruding cell	50 10 100	100 0.1 50	94, 97	94, 96	90, 94	0.94±0.03	0.95±0.03	0.87±0.08
Retracting cell	100 10 500	100 0.1 100	93, 80	84, 90	N/A	0.91±0.03	0.79±0.15	0.80±0.14

Parameters are given as triples (λ, α, n) where λ is the weighted length term,α is the weighted area term, and n is the number of iterations. Parameters A are parameters for the evolution on the morphological closure. Parameters B are for the evolution on the original image when starting from the last round (see 11 for details). For all round cells, the parameters for the evolution on the original image were set to (100, 1, 100) and for the (last round cell) were set to (100, 0.1, 150) and the parameters for morphological closure when working only from the second round cell were set to (100, 0.5, 200). The next 3 columns (labelled Sens) highlight the (sensitivity, specificity) pairs for each of the processing runs All values are given as percentages. The final 3 columns give the mean and standard deviation of the dice coefficients across all frames.

### A. Symmetric Spreading Cell

**Figure 3:**
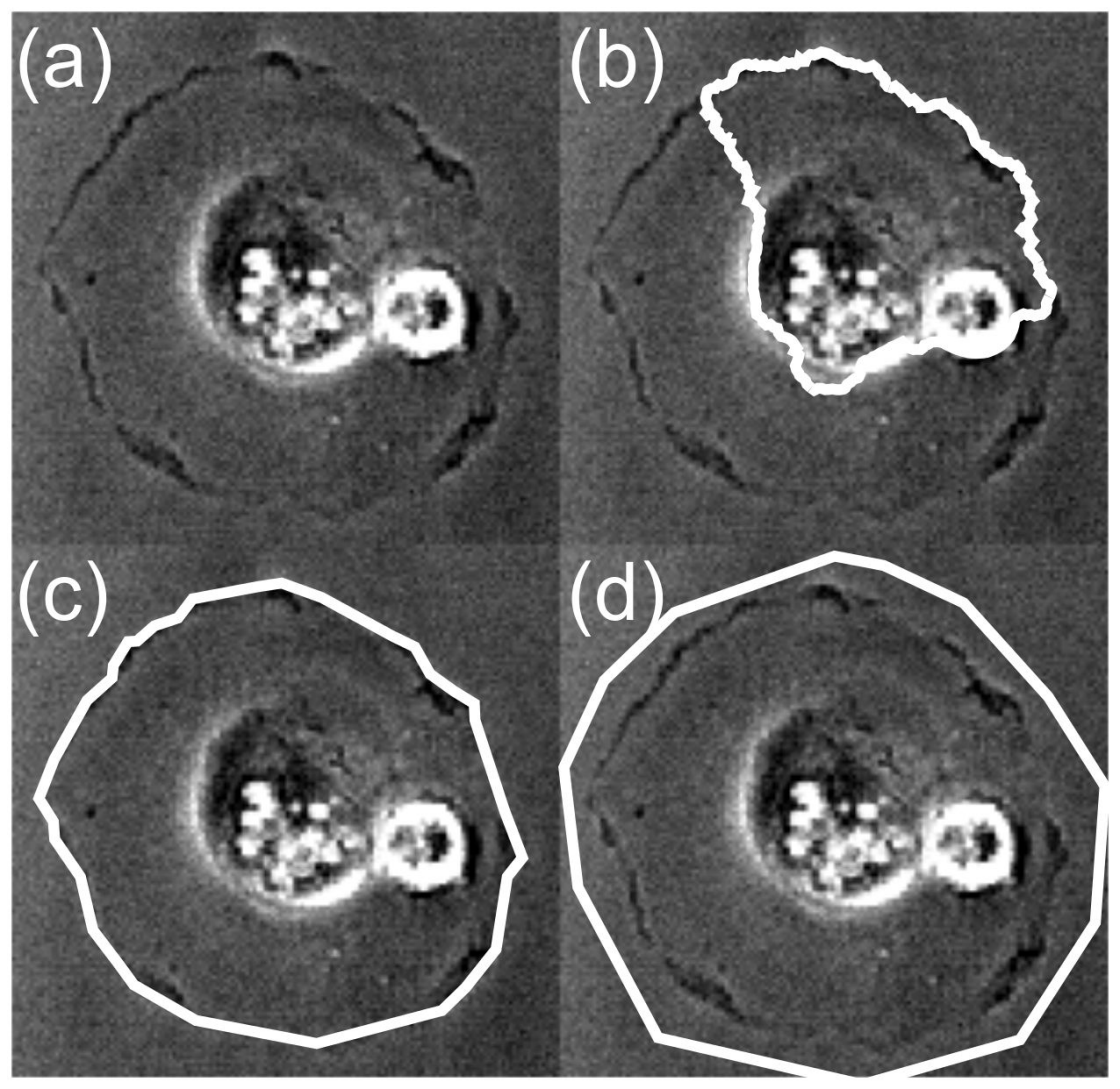
Initialisation for Round cell. a. Microscope image. b. Automatic segmentation result. c. Initialisation contour for CDAC and ABSnake; d. Initialization contour for the Ambühl method (The initialization contour for the Ambühl method should ideally be outside the contour). The cell images in all frames are identical and of the 0^th^ frame (i.e. frame that is segmented by expert). Imaging area: 132×129µm.

As the first real-world dataset, the method was applied to a relatively round cell that does not move significantly (but rather spreads out). See Figure 3. We first observe that the Ambühl procedure for finding the initial cell segmentation does not give satisfactory results (Figure 3), so it fits the requirements for comparison as outlined previously.

**Figure 4:**
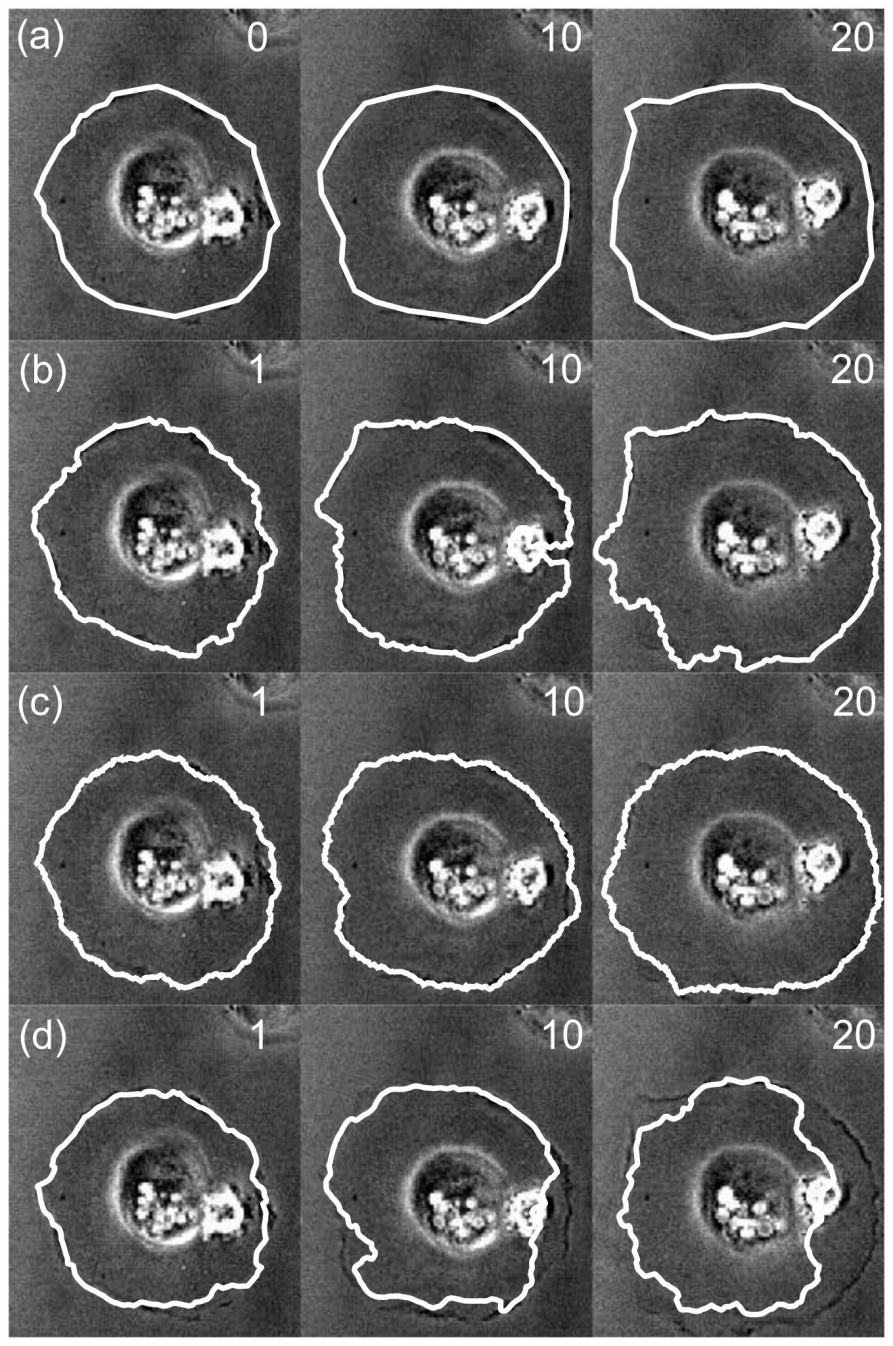
Tracking result for round cell. (a) Expert-drawn outline, (b) output of CDAC, (c) output of Ambühl method, (d) ABSnake. Numbers indicate frame number; frame 0 is the user-drawn initilization, frame 1 is the first automatically-generated outline, and so on.

The contour is thus initialized manually for both methods. CDAC benefits from the initial contour being as close to the outline as possible, whereas the optimal initialization for the Ambühl method is to have the contour slightly outside the cell boundary. For CDAC, the tracking was done for one value of κ (0.02) whereas the tracking was done many times for the Ambühl method, using different combinations of parameters to obtain the highest possible dice coefficient after 20 frames. The value of κ = 0.02 is a good general starting value, after which the value may be reduced if it results in an improved segmentation. The specific parameters are given in Table 1. The parameters for the CDAC and ABSnake methods are given in Table 2 and Table 3 and are the same for all results given in this paper unless explicitly stated otherwise. The tracking results are shown (Figure 4) for the single run of CDAC and the best possible run of the Ambühl method.

**Table 2:** Default parameters for the CDAC method (all data series).

**Parameter**	**Description**	**Typical Value**
*R*	Extrusion search radius	16
*κ*	Energy penalty for extrusion	2×10^-2^

**Table 3:** Parameters for the ABSnake method.

**Parameter**	**Typical value(s)**
Distance Search	100
Displacement	[0.1 - 2.0]
Threshold distance	100
1/α	[0.5 - 2.0]
β	[0.5 - 2.0]
Multiplication factor	0.99

This method is adaptive and automatically selects certain parameters within a pre-specified range. These parameters are given as [*minimum* – *maximum*].

**Figure 5:**
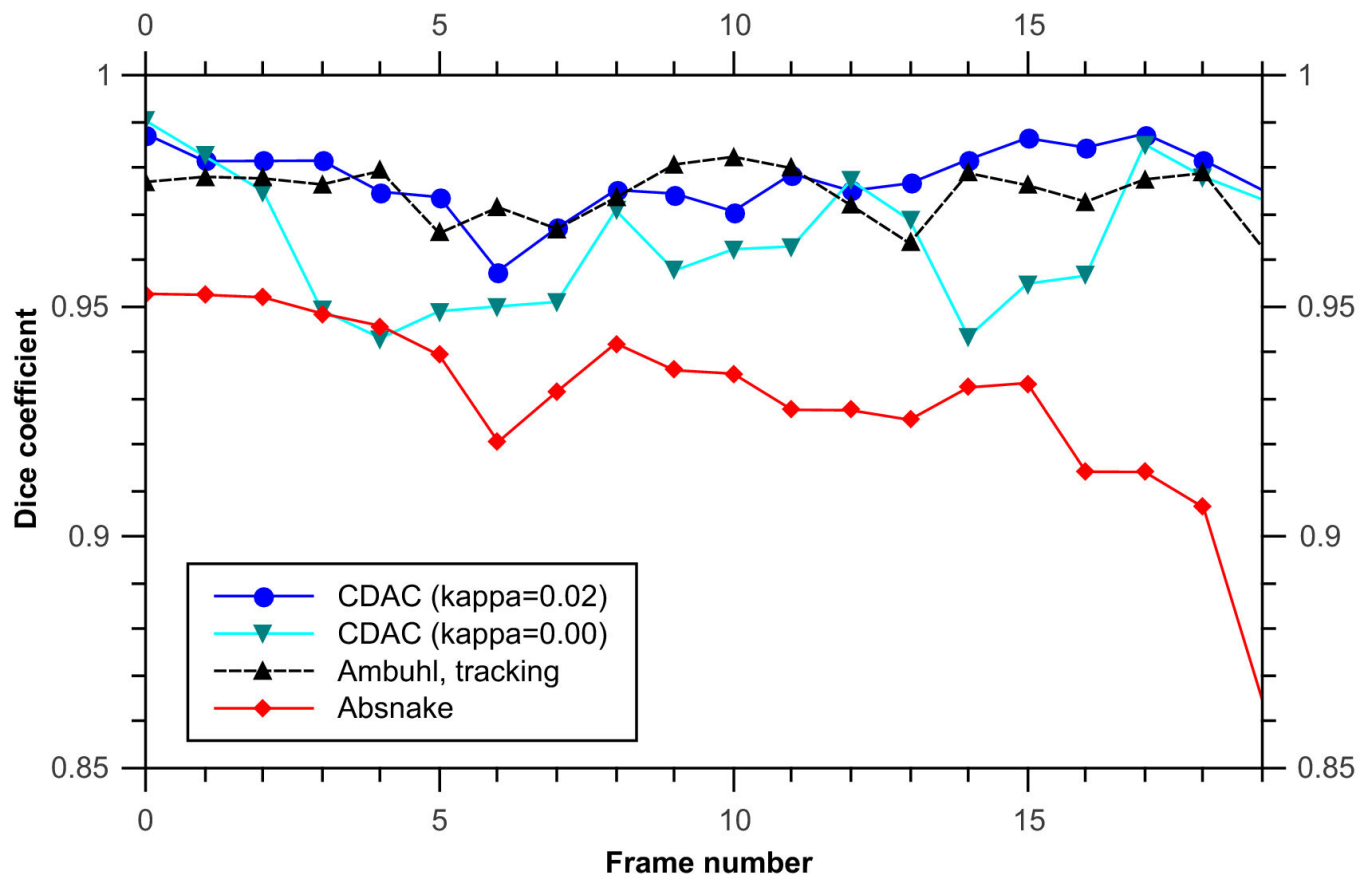
Dice coefficient for tracking symmetric spreading cell. CDAC and the Ambühl method offer similar performance but the performance of ABSnake drops considerably. A noticeable effect is that the dip in quality at frame 6 is shared by both CDAC and ABSnake. However, CDAC recovers quickly. In addition, the coefficient for a value of 0 for κ is given, showing that the performance is not as good as κ=0.02.

In this data set, when considering the dice coefficient, using the Ambühl method in tracking mode gives good results, and CDAC performs similarly (Figure 5). The mean and standard deviation for the dice coefficient for CDAC, Ambühl, and ABSnake were 0.98±0.02, 0.97±0.3, and 0.91±0.5, respectively. This shows that, as can be seen, CDAC and Ambühl are both higher and more stable than ABSnake, with CDAC being slightly more stable in this case. This apparent similar degree of performance could partly be due to the fact that the cell area is relatively large and deviations from the boundary are likely to not overly influence the dice coefficient. It could also be due to the fact that the cell does not move significantly so the outline for the current frame is a good approximation to the outline for the next frame. Thus, for a symmetric spreading cell it was found there was little significant difference between the performances of the methods.

 A possibility arises here: perhaps the slightly better performance of CDAC for these data is due to the data simply being better suited for parametric snakes. If this were the case, other parametric snake methods would also provide better performance than the Ambühl method. As will be seen, however, this is not the case. As a representative snake method, the ABSnake method (as described previously) is used. The results of running the ABSnake method on the latter data set are also shown in Figure 4. As can be seen, the parameters that give the best fit for the initial segmentation (frame 1) fail to provide a good fit for the whole data series. Lowering the internal energy so as to be able to fit into the protrusion causes a highly irregular and numerically unstable curve, whereas raising it results in the outline being ‘too smooth’ to capture fine details. Both approaches ultimately result in loss of the cell outline. In the rest of this paper, the results from ABSnake are also shown alongside CDAC and the Ambühl method for a baseline comparison.

### B. Stationary cell extruding pseudopods

Astrocytes often produce dendritic extrusions as they survey their environment, e.g. direct communication with other cells or when they phagocytose cellular debris following cell death. We are interested in seeing how well both of the algorithms perform on such a cell.

**Figure 6:**
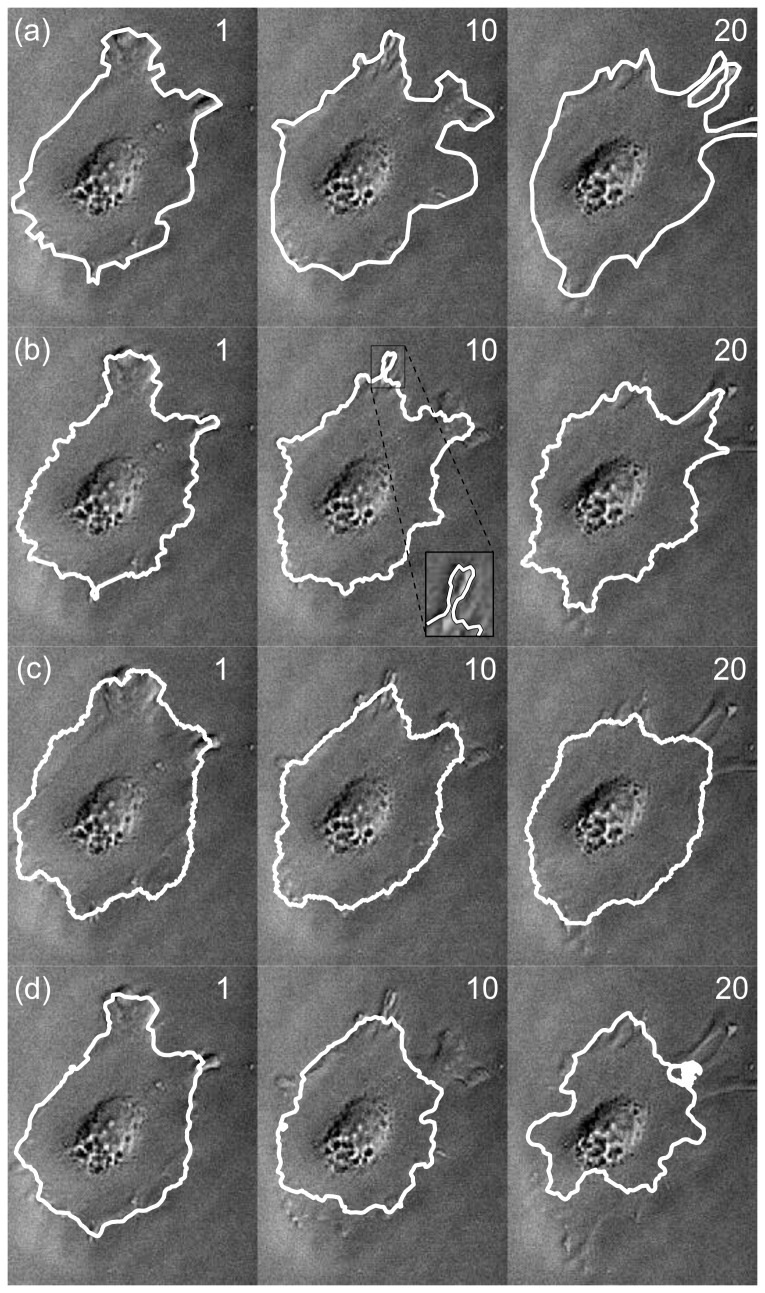
Astrocyte extruding pseudopods. As can be seen, the cell extrudes and retracts projections that are often very low-contrasted. (a) Expert-drawn segmentations, (c) output segmentation of CDAC, (c) output of Ambühl method, (d). ABSnake. As before, numbers indicate frame number. The oval-shaped object in the center is the cell nucleus. What appears to be a crossover in frame 10 of CDAC is actually a point where the contour gets close to itself (inset; magnified and rendering of boundary thinned so this can be more clearly seen). Imaging area: 159×203µm.

The first two methods (Ambühl and CDAC) appear to produce a good segmentation however, with a dice coefficient of above 0.9 for the duration of the whole 20 frames. However, ABSnake fails to lock on to the cell boundary, as is visibly apparent in the tracking images (Figure 6). Its dice coefficient decreases monotonically with increasing frame number.

**Figure 7:**
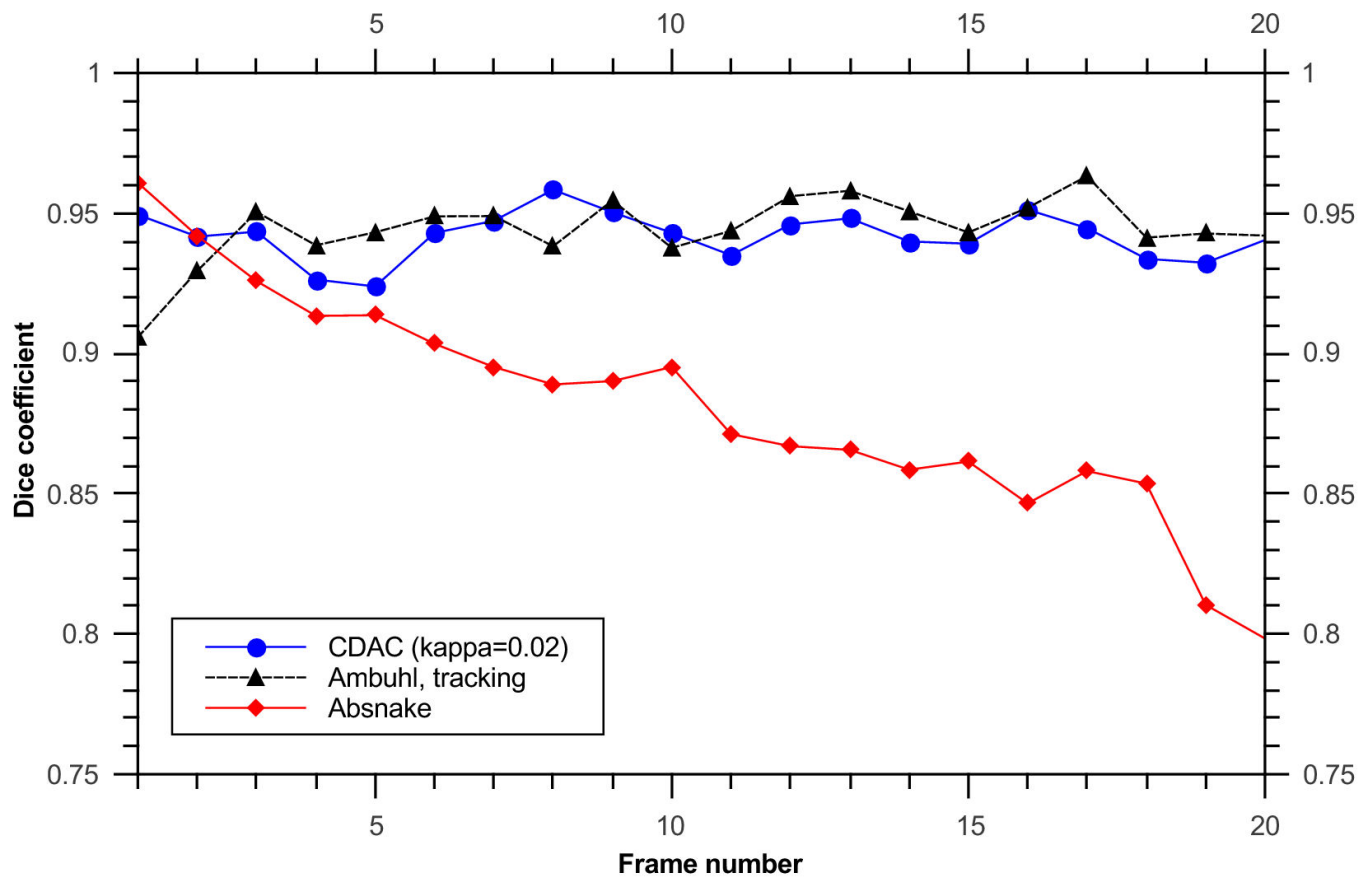
Dice coefficients for tracking a cell with extruding pseudopods.

The dice coefficients of the tracking algorithms, as before, are given in Figure 7. The mean and standard deviation for CDAC , Ambühl, and ABSnake were 0.94±0.03, 0.95±0.03, and 0.87±0.08, respectively.

### C. Cell with retracting protrusion

**Figure 8:**
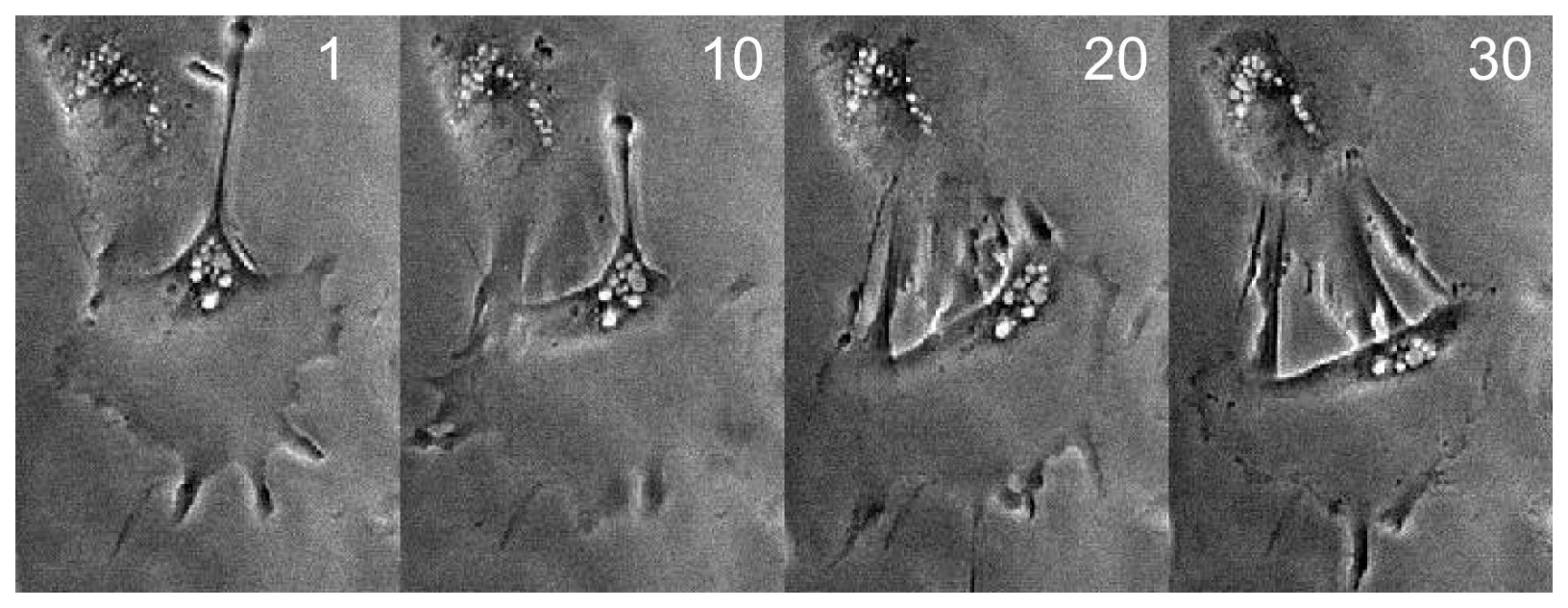
Cell with retracting protrusion. Numbers in top right of frames indicate frame number. Imaging area: 159×239µm.

**Figure 9:**
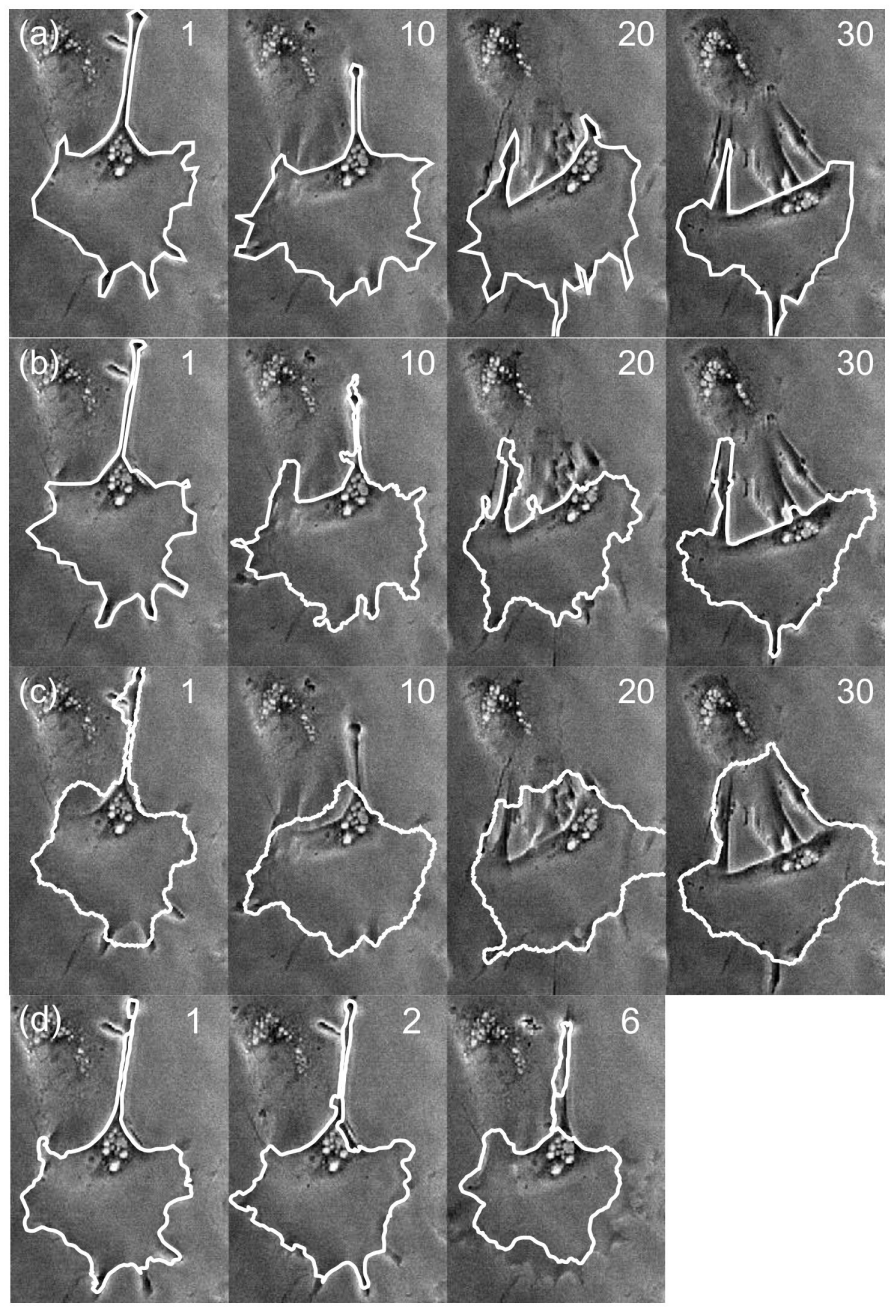
Tracking results for cell with retracting protrusion. (a) Expert-drawn outlines, (b) output of CDAC, (c) Ambühl method, (d) ABSnake. Note that for the ABSnake method, only 6 frames are shown. This is due to the fact that the algorithm diverges in vertex number after frame 6 and thus further tracking is not possible.

Next, the Ambühl method was applied to phase-contrast imagery of an hNT astrocyte both moving and visibly retracting a very thin protrusion (Figure 8 & Figure 9).

**Figure 10:**
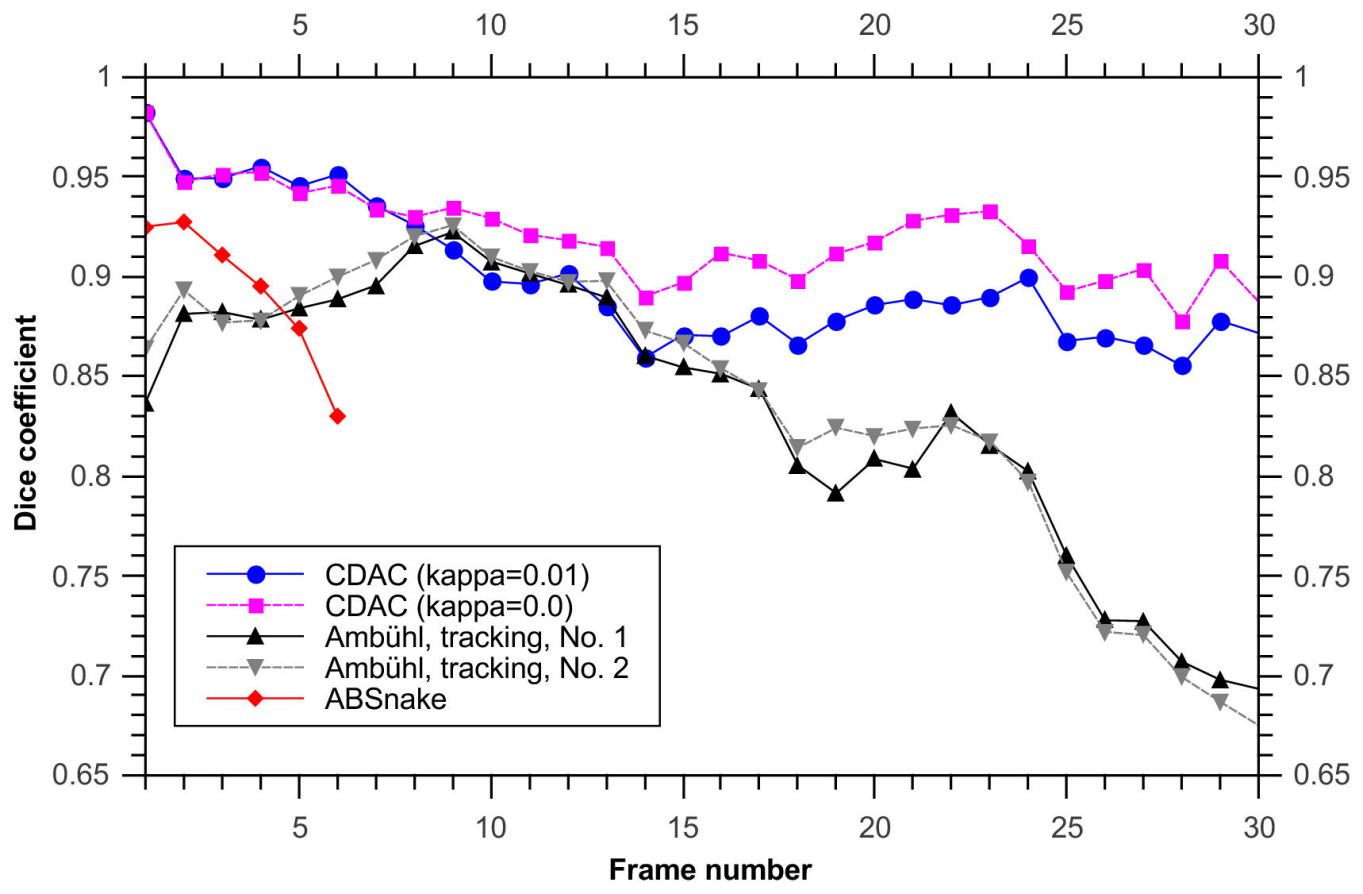
Dice coefficient for tracking cell with retracting protrusion. The two best results from the first two algorithms are shown (For the Ambühl method they are labelled No. 1 and No. 2; parameters given in Table 1. The rapid drop in the quality of the ABSnake-produced segmentation is apparent immediately before vertex divergence.

It is important to note here that the initial high dice coefficient of the CDAC method (Figure 10) is simply due to the expanded initialization required by the Ambühl method. After both methods converge on the boundary, the dice coefficients become similar. The mean and standard deviation for CDAC 1 & 2, Ambühl 1 & 2, and ABSnake were 0.91±0.03, 0.92±0.03, 0.79±0.15, 0.80±0.14, and 0.87±.02 respectively. The two best runs of the Ambühl algorithm are plotted (parameters in Table 1). As can be seen, naive implementation of the Ambühl method (i.e. tracking based on previous frame) has a noticeable decay in performance. While CDAC also suffers from decay, it is able to ‘rebound’ to the correct outline.

The possibility that this relatively low dice coefficient could be due to the thin protrusion of the cell was considered. Thus, we also initialized the Ambühl method around the cell body only, ignoring the protrusion. However, it was observed that the results were similar.

**Figure 11:**
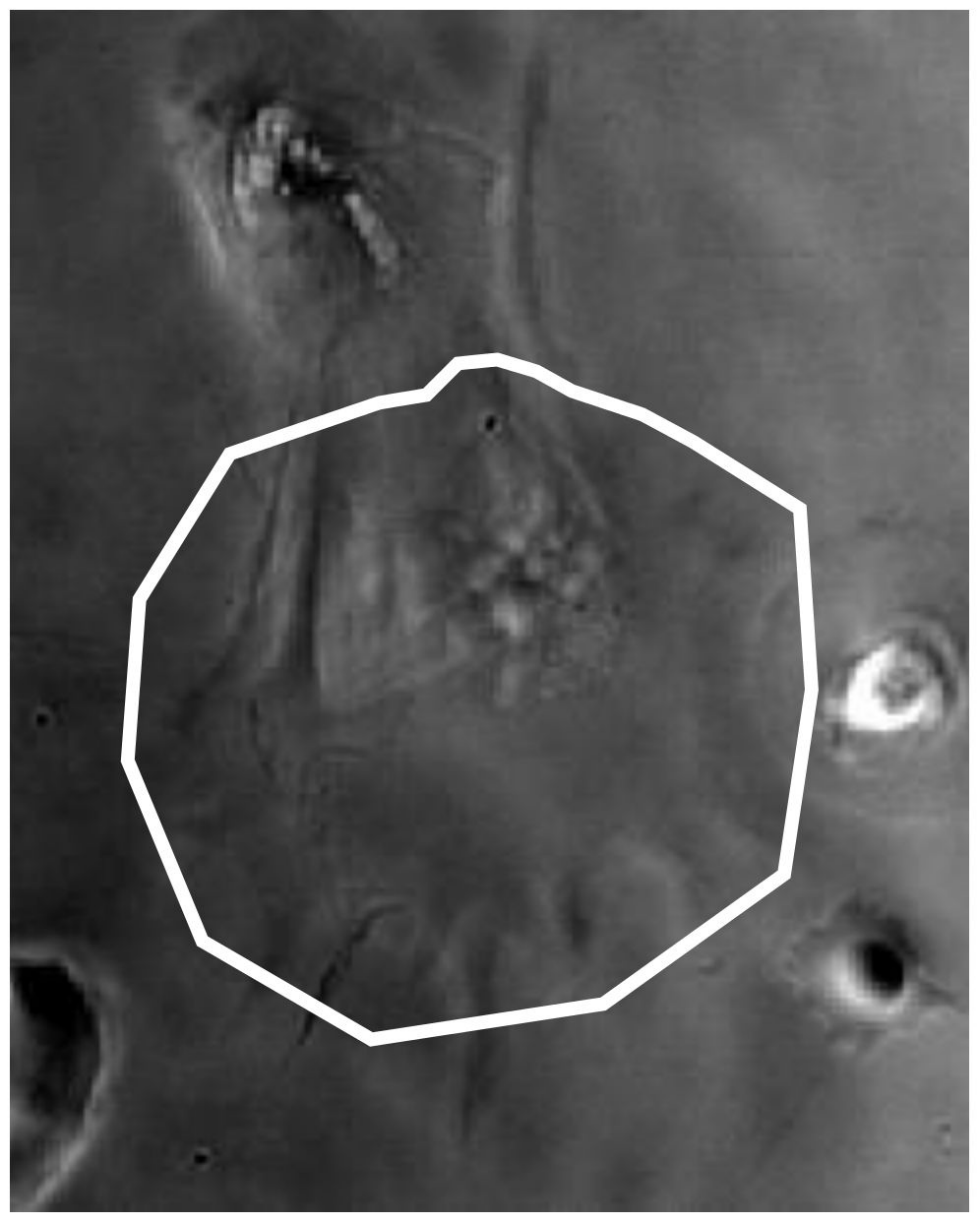
Valid initialization for cell body for all 30 frames. The frames have been superimposed to show that the outline encapsulates the cell body relatively tightly. As indicated in the text, the image is a superposition of all 30 frames.

The possibility was also considered that this decay in performance could be remedied by using the Ambühl method in ‘re-segmentation mode’ i.e. initializing from the same manually-drawn contour at each frame, or a new manually-drawn contour every certain number of frames, as opposed to using the contour from the previous frame. First, an attempt was made to draw a contour that would encapsulate the entire cell over the course of 30 frames (Figure 11). An important drawback of this method is that it is not possible in real-time; all cell outlines have to be gathered before it can be done.

**Figure 12:**
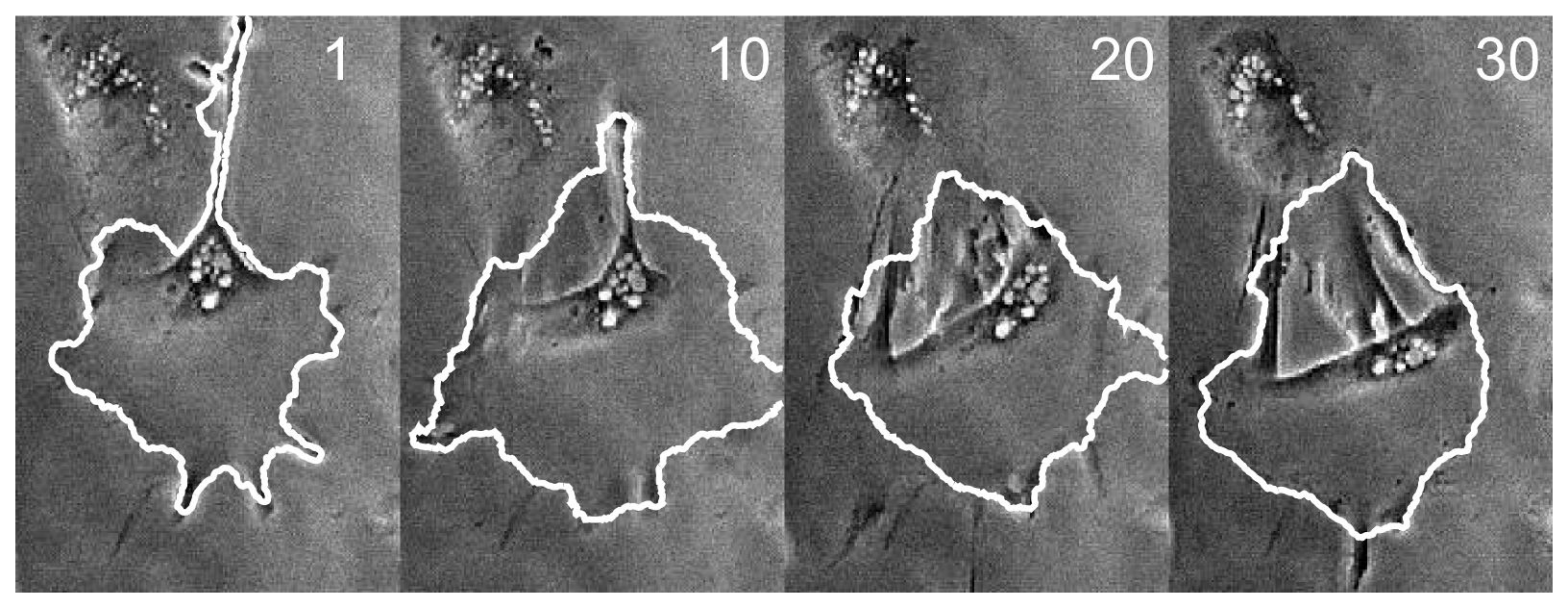
Segmentation results for re-initializing the contour at each frame.

**Figure 13:**
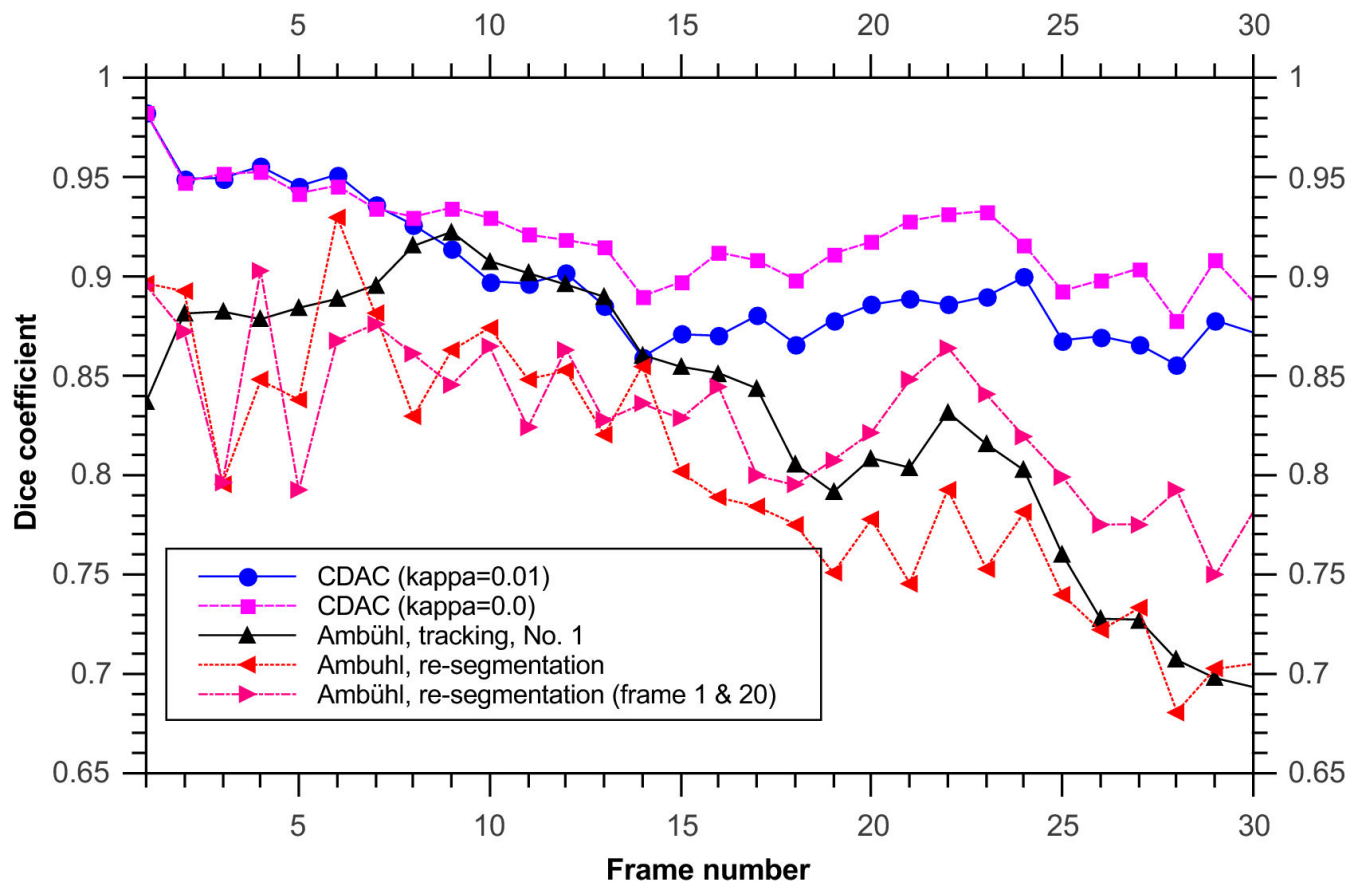
Dice coefficient for tracking cell using re-segmentation from user-drawn contour at each frame. Shown are plots for our method and Ambühl’s method in both tracking mode (as in the previous example) and re-segmentation at each frame. Since the two best runs in tracking mode are similar, only the first is shown for clarity.

It was observed that, contrary to expectations, the segmentation quality was similar to simply initializing from the previous contour, (Figure 12 and Figure 13) although it should be noted that the produced outlines are different. 

To obtain the highest possible performance, it would seem the best option would be to re-initialize the contour once the dice coefficient starts to drop. To do this, the contour was re-initialized manually at frame 20 and then the algorithm was run again with multiple parameters in order to select the best fit. Care must be taken to choose where to re-initialize. If some frames are inherently problematic (for example, very irregular shape or very low contrast), re-initializing at those frames would provide little benefit as the segmentation algorithm would lose its lock regardless. To avoid an unfair comparison, the frame at which re-initialization is done is as late as possible (to avoid troublesome areas) without letting the dice coefficient persist at a low value for too long. As can be seen, frame 20 is a reasonable choice for this. This choice biases the comparison slightly in favor of the Ambühl method. The results are also plotted (Figure 13). The mean and standard deviation for the 2 CDAC and 3 Ambühl methods were 0.98±0.02, 0.97±0.3, 0.79±0.15, 0.77±0.16, and 0.82±0.13, respectively. It can be seen that though the quality is now much better, the segmentation produced by the Ambühl method still lags behind what is produced by CDAC, even when still running from the segmentation produced in frame 0.

The analysis of the sensitivity and specificity (see IV Results) given in Table 1 roughly reflect what is seen with the dice coefficient. Both the sensitivity and specificity of CDAC is remarkably higher than either of the two other methods in the retracting cell. The highest sensitivity for the Ambühl method is only achieved when two segmentations are done yet it is still lower than that produced by CDAC.

## Discussion

A cell outline tracking algorithm (CDAC) that utilizes information about how cells move to produce a high-quality segmentation was presented. Actin-based cellular motility proceeds by pushing against the membrane. By restricting each vertex on the cell outline to only move in the normal direction, the search space for updating each vertex is simplified, allowing a global optimization method to be used. We have compared CDAC with a state-of-the-art active contour method (the Ambühl method), using an error metric based on segmentation area. The comparison was conducted using three different data sets, showing different behaviours. We have shown that despite the simplicity and computational efficiency of CDAC, it produces results of quality at least as high as the Ambühl method.

In choosing a segmentation method for cells in general, many considerations have to be made such as: The types of cells and their shapes; the number of frames between which manual re-adjustments can be made; the decision to use manual vs. automatic initialization and the time available for the segmentation process are all critical considerations. In addition, the complexity of the method must be taken into account; methods that render too complex might not lend themselves to customization for specific situations. It is also important to consider the potential of the method for tracking cells in more complicated environments such as those involving a large number of cells. While the Ambühl algorithm [23] is a well-performing general-purpose segmentation method, situations arise where methods better suited to the problem domain (CDAC) can provide further improvements, when considering certain properties of the produced cell boundary. A brief discussion of these situations follows.

The mathematical formulation of CDAC was given based on parametric active contours. The results of the method on the various data sets illustrates why various design choices were made. Adaptive subdivision allows global optimization to be efficient and global optimization provides a firm basis for the next iteration of the subdivision. For the cell extruding pseudopods, the Ambühl method is conservative, localizing the general cell outline but failing to identify the extrusions. CDAC, on the other hand, fits into protrusions well. This ability of CDAC to deal with irregular shapes is sometimes detrimental; in the frame sequence of the round cell CDAC erroneously latches on to some debris. However, it 're-corrects' itself in the next frames, something that would not be possible with a local optimization procedure.

In the cell with retracting protrusion, the cell interacts with another cell, but it still maintains a strong edge near the top of the cell which the Ambühl method fails to localize but which CDAC identifies correctly. This is an example of an issue where re-initialization, if it is to be used, must be done carefully by the user to not overlap the second cell; otherwise the contour will settle down on the other cell's contours and not reach the strong edge which is between the cells. However, even with re-initialization, the cell may move in way so that a tight-fitting contour in one frame becomes a contour that encloses both cells several frames later. This necessitates more frequent manual segmentation and requires care that only the desired cell be enclosed. CDAC does not appear to suffer from any of these difficulties. However, one drawback of CDAC is that the contour initialization must be relatively accurate, or at least close enough that the cell boundary can be obtained from the contour in a normal extrusion step.

Trade-offs such as this in choosing how often to manually re-initialize the cell contour blur the boundary between automatic and semi-automatic segmentation methods, and methods based on initializing the contour from the previous frame or initializing from some manually-drawn approximation that is valid over a wide range of frames. The Ambühl method performed considerably better when used in the semi-automatic mode of operation versus the former, but this requires more manual intervention and is still outperformed by CDAC. On the final data set with a very irregular cell, CDAC performed better than the Ambühl method even when only one manually-drawn contour was required as opposed to the two such contours required for the Ambühl method.

In CDAC, the energy function is directly related to the shape of the curve and its effects can be seen in the evolution of the curve. Thus fine-tuning of the energy function for different problems becomes possible. This is an advantageous property and some level set methods also have tuning parameters that separately capture the relative weight between the shape/geometry terms and image fitting.

However, one of the main advantages often given for using a geometric method is that multiple cells can be tracked without additional penalty. However, as has been demonstrated, in the geometric method multiple cells are often confused with each other, with one cell sometimes being identified as two separate cells, or two separate but highly-interacting cells being identified as one. CDAC does not suffer from these problems, as can be seen in the third image series where the cell interacts with its neighbor. Further, the level set method is sufficiently more computationally expensive than the parametric method that even with an imaging field saturated with cells, requiring up to two orders of magnitude more computational time, the parametric method would still perform faster. Additionally, in the parametric method the concept of a very thin boundary shared by two cells has meaning; something that is not easily attainable with the geometric method (and many other active contour methods) [58] since it relies on the zero isoline of a single distance-to-boundary function (the level set itself). This information can be used to the advantage of segmenting algorithms. For example, when two cells approach each other, the boundary between them can be identified and preserved by the algorithm, something that is being explored for future work.

Finally, computational considerations are very important in real-time high-throughput data-collection workflows. The run-time of the Ambühl method averaged about 400 seconds per run (see the section ‘Results’), whereas for CDAC it is on the order of less than two seconds per run. This difference of two orders of magnitude is mainly attributable to the level-set update procedure which is very costly, not to any specific implementation details. It is important to emphasize again that the parameters chosen for the Ambühl method were based on what parameters gave the best segmentation, whereas for CDAC the default parameters were chosen initially and then never changed. Thus, in practice it took far longer to use the Ambühl method on each data set than to use CDAC. The selection procedure for the parameters for the Ambühl method took a large number of combinations into account, resulting in each run of the Ambühl method taking about 20 hours in total. This is in contrast with the several seconds required for each run of CDAC.

It is for these reasons, in addition to the reliability offered by the insensitivity to parameters and rapid deployability, that we believe CDAC is better suited to segmentation problems involving irregular cells in suboptimal imaging conditions.

### Supplementary Material

Source code for CDAC may be found on https://github.com/anj1/cdac. All data used to produce the figures in this paper have been provided as supplemental files.
