# Correction: A Cell Derived Active Contour (CDAC) Method for Robust Tracking in Low Frame Rate, Low Contrast Phase Microscopy - an Example: The Human hNT Astrocyte

**DOI:** 10.1371/journal.pone.0100070

**Published:** 2014-06-05

**Authors:** The PLOS ONE Staff

There are errors in the Author Contributions. The correct contributions are: Conceived and designed the experiments: ANJ. Performed the experiments: ANJ. Analyzed the data: ANJ CPU. Contributed reagents/materials/analysis tools: ESG. Wrote the manuscript: ANJ.
